# A Printed Organic Circuit System for Wearable Amperometric Electrochemical Sensors

**DOI:** 10.1038/s41598-018-24744-x

**Published:** 2018-04-23

**Authors:** Rei Shiwaku, Hiroyuki Matsui, Kuniaki Nagamine, Mayu Uematsu, Taisei Mano, Yuki Maruyama, Ayako Nomura, Kazuhiko Tsuchiya, Kazuma Hayasaka, Yasunori Takeda, Takashi Fukuda, Daisuke Kumaki, Shizuo Tokito

**Affiliations:** 1 Research Center for Organic Electronics (ROEL), Yamagata University, 4-3-16 Jonan, Yonezawa Yamagata, 992-8510 Japan; 2 Functional Polymers Research Laboratory, Tosoh Corporation, 1-8 Kasumi, Yokkaichi, Mie 510-8540 Japan

## Abstract

Wearable sensor device technologies, which enable continuous monitoring of biological information from the human body, are promising in the fields of sports, healthcare, and medical applications. Further thinness, light weight, flexibility and low-cost are significant requirements for making the devices attachable onto human tissues or clothes like a patch. Here we demonstrate a flexible and printed circuit system consisting of an enzyme-based amperometric sensor, feedback control and amplification circuits based on organic thin-film transistors. The feedback control and amplification circuits based on pseudo-CMOS inverters were successfuly integrated by printing methods on a plastic film. This simple system worked very well like a potentiostat for electrochemical measurements, and enabled the quantitative and real-time measurement of lactate concentration with high sensitivity of 1 V/mM and a short response time of a hundred seconds.

## Introduction

Wearable sensor devices enabling continuous real-time monitoring and analysis of biological information from the human body are promising in the fields of sports, healthcare, and medical applications^1–3^. A variety of biomarkers in body fluids, such as perspiration and saliva, can be continuously detected by a wearable electrochemical sensing system, which enables *in situ* analysis of physiological signals^4,5^. An enzyme-based amperometric sensor is one of the most important electrochemical sensors owing to the high selectivity and the connectivity to information technologies. So far, the quantitative measurement of metabolites in human fluids, including lactate^6^, glucose^7^, and uric acid^8^, have been carried out using enzyme-based amperometric sensors and conventional potentiostat^9^ systems. A potentiostat is the electronic hardware which controls the three-electrode cell for electrochemical experiments and possesses two functions: (1) maintaining the potential of the working electrode (WE) at a constant level with respect to the reference electrode (RE) by adjusting the current at a counter electrode (CE), and (2) converting the current at the working electrode to the voltage by a transimpedance amplifier with a high gain. Hence, both the enzyme-based amperometric sensors and the potentiostat need to be integrated in the wearable devices.

Organic thin-film transistors (OTFTs) have potential for realizing ultra-thin, lightweight^10^, and flexible^11^ circuit components of the potentiostat for wearable sensor devices owing to their advantages such as the small Young’s modulus, biocompatibility, and the processability of direct printing onto plastic films. Printability is an attraction of OTFTs because organic materials can be dissolved in organic solvents, which enables roll-to-roll manufacture of large-area devices on flexible substrates^12,13^. So far, OTFTs have been utilized as amplifiers for potentiometric electrochemical sensors, which is called extended-gate type OTFTs^14^.

Although the potentiometric measurement is applicable to enzymatic sensors, it exhibits an irreversible and slow response in several minutes, which is not suitable to real-time sensing with wearable devices. On the other hand, the amperometric measurement based on enzymatic sensors exhibits a reversible and fast response in several tens of seconds^4^. OTFTs have never been utilized for amperometric sensors since the three-electrode cell requires integrated circuits rather than the simple extended-gate type OTFTs.

Here we demonstrate a novel flexible and printed organic circuit system for wearable amperometric electrochemical sensors, implemented with two OTFT-based negative-feedback inverters. The inverters employed pseudo-CMOS design for obtaining rail-to-rail operation and low output impedance, and consisted of the OTFTs based on a blend of a small molecular p-type semiconductor and polystyrene (PS) for the active layer. The first inverter was utilized to maintain the potential at the working electrode (WE) at a constant level with respect to the reference electrode (RE). The second inverter was utilized to convert the current at the WE into voltage with a tunable gain of 10^6^–10^7^ V/A. A lactate sensor with a lactate oxidase membrane was used as the WE. Real-time, quantitative measurement of lactate concentration was successfully demonstrated by the developed system, showing the response time of a hundred seconds and the sensitivity of 1 V/mM in a lactate concentration range of 0–0.5 mM.

## Results and Discussion

### Three-Electrode Circuit System for Amperometric Analysis

**Figure 1:**
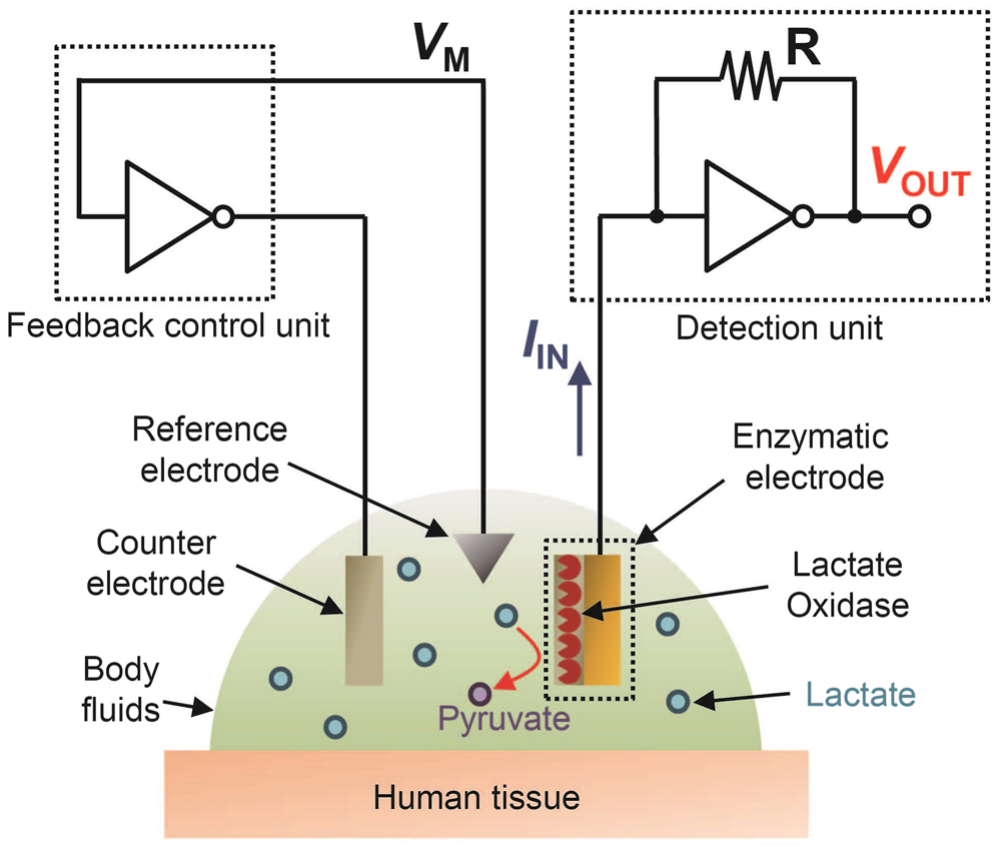
A three-electrode circuit system for wearable amperometric electrochemical sensors. The system is composed of a feedback control unit, a detection unit, and a three-electrode cell with an amperometric electrochemical sensor (e.g. lactate sensor) as a working electrode.

Figure 1 displays the configuration of the developed system for amperometric sensing, based on three components: a three-electrode electrochemical cell, a feedback control unit, and a detection unit. The lactate sensor in the three-electrode cell has an immobilized lactate oxidase on the electrode for selective detection of lactate in body fluids. The current that is generated from the lactate sensor electrode (working electrode: WE) by enzyme reaction varies linearly with the concentration of lactate in the cell (at low concentration region), according to the Michaelis-Menten equation. The detection unit, which comprises an inverter and a resistor, converts current (*I*_IN_) to voltage (*V*_OUT_) by a transimpedance amplifier with predefined gain. Assuming that the open-loop gain of the inverter is high enough, *V*_OUT_ is given by

1 $${V}_{{\rm{O}}{\rm{U}}{\rm{T}}}={V}_{{\rm{M}}}-R{I}_{{\rm{I}}{\rm{N}}}.$$

Here *V*_M_ is the switching voltage of the inverter. In the detection unit, the potential of the input terminal, namely the WE, is kept at *V*_M_ by low input impedance. The feedback control unit comprises an inverter only, and is used to keep the potential of the reference electrode (RE) at *V*_M_. This unit ensures, at the same time, that there is no current flow through the RE, which is necessary for the RE to function as a potential standard properly. Either CMOS, PMOS or NMOS inverters can be used for this system as long as they exhibit high open-loop gain, small variation in *V*_M_, and low output impedance.

### Fabrication and Characterization of Lactate Sensor

**Figure 2:**
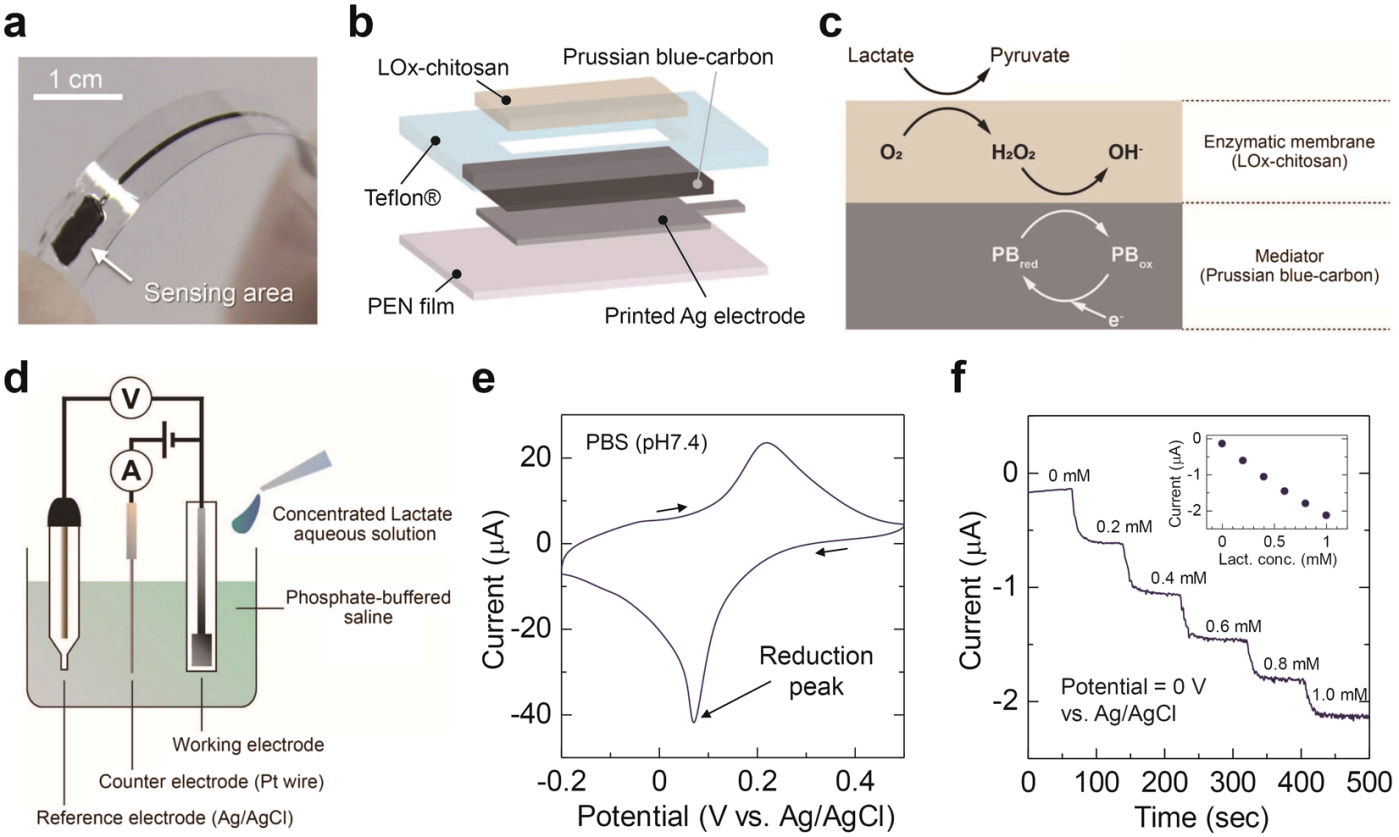
Structure and electrochemical characteristics of the lactate sensor. (**a**) Photograph of the fabricated lactate sensor electrode. The sensing area was 15 mm^2^. (**b**) Schematic diagram of the lactate sensor electrode. (**c**) The principle of lactate sensing. (**d**) Schematic representation of amperometric measurement with a commercial potentiostat. (**e**) Cyclic voltammogram of the lactate sensor in phosphate-buffered saline (PBS). Scan rate was 20 mV/s. (**f**) The amperometric responses of the lactate sensor. Potential of the lactate sensor electrode was set to 0 V vs. Ag/AgCl reference electrode.

The photograph and schematic structure of the lactate-sensitive working electrode is shown in Fig. 2a,b. Prussian blue (PB)^15^ was chosen as a mediator, and a PB-carbon graphite composite paste^16^ was coated onto the inkjet-printed Ag electrode surface. A fluoropolymer bank layer was formed at a periphery of the PB-carbon to define the sensing area. The interconnection part of the Ag film was also encapsulated by a fluoropolymer for prevention of Ag/liquid contact^17^. Finally, a blend of lactate oxidase (LO_X_) and chitosan for the enzyme immobilization was coated onto the mediator layer^18^. Figure 2c represents the principle of lactate sensing. The immobilized LO_X_ selectively oxidizes lactate, and generates pyruvate and H_2_O_2_. The H_2_O_2_ then oxidize the PB from the reduced state (PB_red_) into the oxidized state (PB_ox_). PB_ox_ accepts an electron from carbon graphite and return to PB_red_. These reactions continuously occur under the presence of lactate, and the electric current flows in the direction from the electrolyte to the Ag electrode. Figure 2e shows the cyclic voltammogram of the lactate sensor electrode in a three-electrode cell (Fig. 2d) with a commercial potentiostat. The peak of reduction current was observed at a potential of +0.07 V vs. Ag/AgCl reference, approximately corresponding to the values from previous reports^19^. Figure 2f shows the amperometric responses at a potential of 0 V vs. Ag/AgCl. When concentrated lactate solution was added into the cell, the current changed stepwise in several tens of seconds. The current amplitude was proportional to the lactate concentration with the sensitivity of 2 µA/mM in the range of 0–1 mM (inset in Fig. 2f). The average sensitivity of 5 samples was 1.9 ± 0.2 µA/mM (maximum: 2.2 µA/mM, minimum: 1.7 µA/mM). The sensitivity was stable under five repetitive measurements (see Supplementary Figure S1).

### Structure and Electrical Properties of OTFT Devices

**Figure 3:**
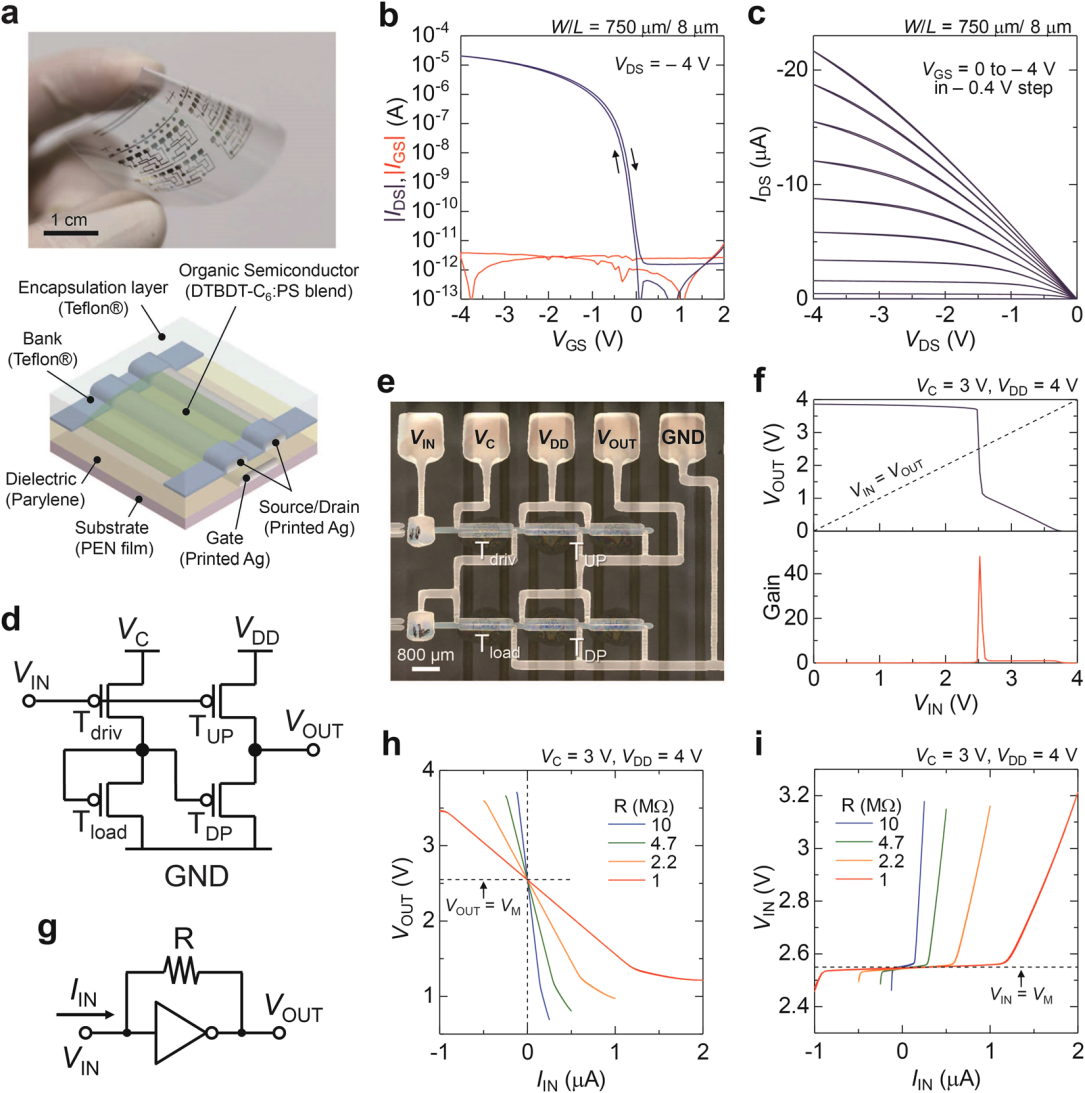
Structure and electrical properties of the printed organic semiconductor devices. (**a**) Photograph (top) and schematic structure (bottom) of the OTFTs. (**b**) Transfer curves and (**c**) output curves of the OTFT. (**d**) Circuit diagram and (**e**) optical microscope image of the pseudo-CMOS inverter. (**f**) Static input-output characteristics of the inverter. Output voltage (*V*_OUT_) and small-signal gain (|d*V*_OUT_/d*V*_IN_|) as a function of input voltage (*V*_IN_). (**g**) Circuit diagram of the current-to-voltage converter. (**h**) *V*_OUT_ and (**i**) *V*_IN_ of the current-to-voltage converter as a function of *I*_IN_. Value of resistance was set to 1–10 MΩ.

Figure 3a displays the photograph and schematic illustration of the fabricated OTFT devices. All layers except for the gate dielectric were formed by printing processes at process temperatures below 120 °C. The electrodes were fabricated by inkjet printing of a silver nanoparticle ink. A fluoropolymer bank layer, semiconducting layer, and encapsulation layer were printed by a dispenser equipment. In the same way as the lactate sensor electrodes, the OTFT devices were fabricated on flexible poly(ethylene naphthalate) (PEN) films owing to the low process temperature. A fluoropolymer bank layer was used to define the channel width precisely and also to control the crystal growth of the organic semiconductor, which leads to the uniform morphology^20^. A blend solution of 2,7-dihexyl-dithieno[2,3-*d*;2′,3′-*d*′]benzo[1,2-*b*;4,5-*b*′]dithiophene (DTBDT-C_6_)^21^ and polystyrene (PS) was used as the active layer to obtain high mobility and uniform electrical performances^22,23^. Figure 3b shows the transfer characteristics of the OTFT in a saturation regime. The mobility of 1.3 cm^2^/Vs, threshold voltage of −0.25 V, and subthreshold slope of 100 mV/dec were obtained at a low supply voltage of −4 V. According to the output curve in the linear regime shown in Fig. 3c, the semiconductor/electrode contact was ohmic rather than schottky.

Figure 3d,e shows the schematic structure and optical microscope image of the inverter circuit with pseudo-CMOS design configurable with p-type TFTs only^24^. The pseudo-CMOS inverter comprises a depletion-load inverter^25^ and an output stage for rail-to-rail output and low output impedance. Figure 3f shows the static input-output characteristics of the inverter. Two supply voltages of *V*_C_ = 3 V and *V*_DD_ = 4 V were applied to adjust the switching voltage of the inverter (*V*_M_). Finally, *V*_M_ of 2.6 V and open-loop gain of 50 were obtained.

Figure 3g represents a transimpedance amplifier (*I*-*V* converter) based on a negative feedback inverter. Assuming that the input-output characteristics of the inverter in the vicinity of *V*_M_ is expressed as *V*_OUT_ = *V*_M_ − *A*_open_(*V*_IN_ − *V*_M_), the relation between *V*_OUT_ and *I*_IN_ is represented by the following equation:

2 $${V}_{{\rm{O}}{\rm{U}}{\rm{T}}}={V}_{{\rm{M}}}-\frac{R}{1+\frac{1}{{A}_{{\rm{o}}{\rm{p}}{\rm{e}}{\rm{n}}}}}{I}_{{\rm{I}}{\rm{N}}}.$$

(For details, see Supplementary Figure S2). Consequently, transimpedance gain is given by:

3 $$\frac{d{V}_{{\rm{O}}{\rm{U}}{\rm{T}}}}{d{I}_{{\rm{I}}{\rm{N}}}}=-\frac{R}{1+\frac{1}{{A}_{{\rm{o}}{\rm{p}}{\rm{e}}{\rm{n}}}}},$$

where *A*_open_ is the open-loop gain of the inverter. In the case of a sufficiently high open-loop gain, *A*_open_ >> 1, the transimpedance gain is given by −*R*. The gain in this work was deviated from −*R* by 2%. The gain of 10^6^–10^7^ V/A was obtained at *R* of 1–10 M Ω (Fig. 3h). The voltage at the input terminal, *V*_IN_, was maintained at *V*_M_ in the linear regime of *V*_OUT_, which indicates low input impedance of the transimpedance amplifier (Fig. 3i). For instance, *V*_IN_ was stable against *I*_IN_ of ±1 µA at *R* of 1 MΩ. For a wide range of *I*_IN_, reduction of the output impedance of the inverter should be required. Output impedance of the inverter was 0.1 and 0.3 MΩ at forward and reverse current, respectively.

### Application of The Organic Circuit System to Lactate Sensor

**Figure 4:**
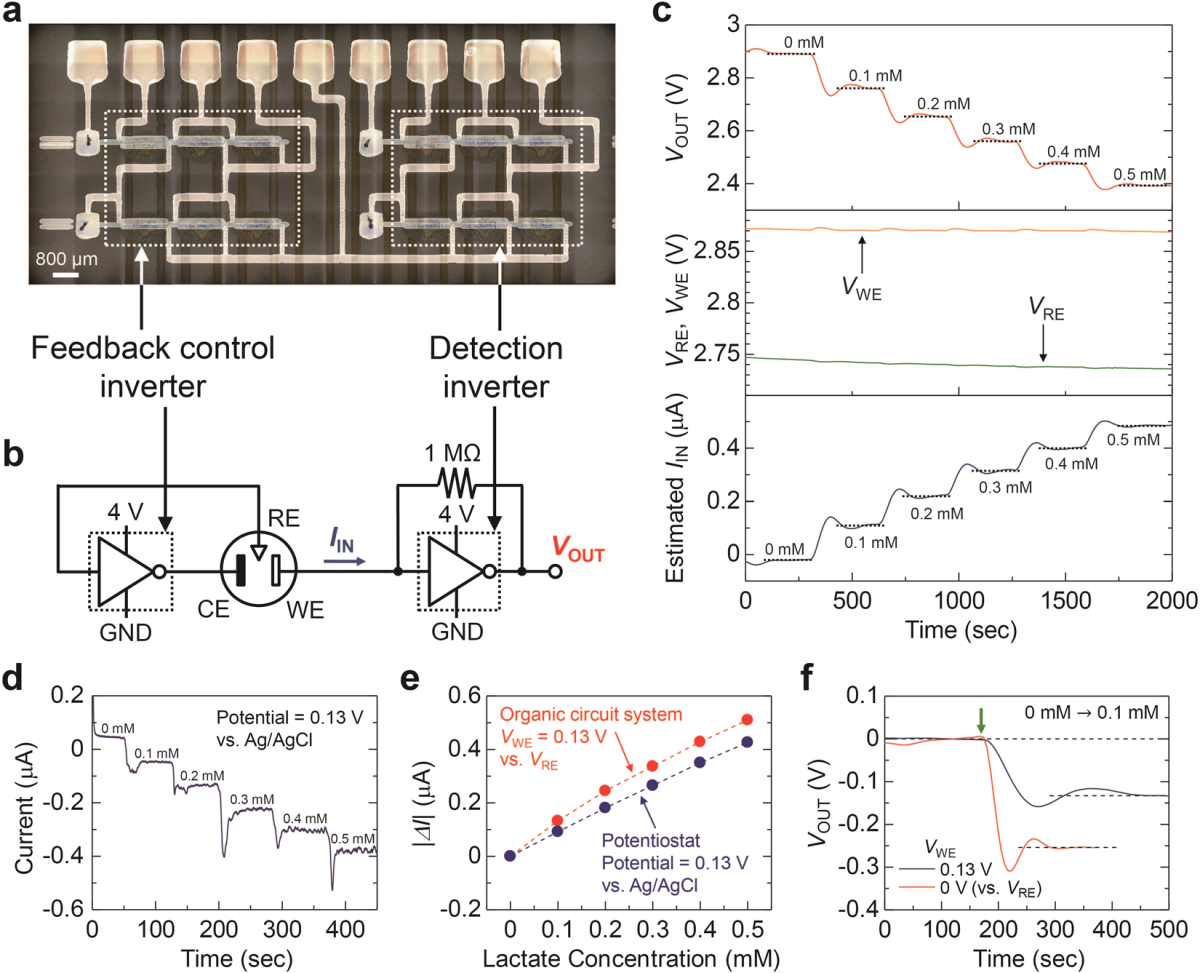
Quantitative measurement of lactate concentration in the three-electrode electrochemical cell using the printed organic circuit system. (**a**) Optical microscope image of the inverter pair. (**b**) Circuit diagram of the system. Control voltage (*V*_C_) and supply voltage (*V*_DD_) of both feedback control inverter and detection inverter was set to 3 V and 4 V, respectively. (**c**) Output voltage (*V*_OUT_), potential of the working electrode (*V*_WE_) and reference electrode (*V*_RE_), and estimated input current (*I*_IN_), obtained from the organic circuit system. Concentrated lactate solution was added every 300 seconds. (**d**) Amperometric responses from a commercial potentiostat. The potential of the working electrode was set to 0.13 V vs. Ag/AgCl. (**e**) Comparison of the absolute values of the change of current (|*ΔI*|) from a commercial potentiostat and the organic circuit system. (**f**) Comparison of *V*_OUT_ from the organic circuit system at *V*_WE_ of 0 and 0.13 V vs. *V*_RE_. The concentration of lactate was changed from 0 to 0.1 mM. The green arrow means the timing of dropping the concentrated lactate solution into the cell.

The amperometric sensing system was demonstrated using the two pseudo-CMOS inverters on the same substrate, shown in Fig. 4a,b. Figure 4c shows *V*_OUT_, potential of the RE (*V*_RE_), potential of the WE (*V*_WE_), and *I*_IN_ estimated by Eq. 2. Responding to the addition of lactate into the cell, *V*_OUT_ changed stepwise in a hundred seconds at lactate concentration of 0–0.5 mM. The obtained sensitivity after the transimpedance amplification was as high as 1 V/mM. The high output voltage of several hundreds of mV can be easily read by analog-to-digital converters for further data processing, logging and wireless communication in near future^26^ Regardless of the concentration of lactate, the *V*_RE_ and *V*_WE_ were maintained at 2.74 V and 2.87 V (close to *V*_M_ of the inverters), respectively. During the measurement period of 2000 seconds, the variations in each voltage were less than 0.01 V. The stability of the *V*_RE_ and *V*_WE_ indicates that the feedback control unit is working very well in this system.

At last, we discuss the influence of the voltage difference, *V*_WE_ − *V*_RE_ = 0.13 V, which was equivalent to the difference in *V*_M_ of the two inverters (see Supplementary Fig. S3). According to the estimated *I*_IN_, the sensitivity of the developed lactate sensing system before the transimpedance amplification was 1 µA/mM, which was approximately half of that of a commercial potentiostat in Fig. 2f. In addition, the response time was two or three times longer than that with a potentiostat. The deteriorations in the sensitivity in current and response time were attributed to the difference in *V*_M_ of the inverters. The *V*_WE_ should be close to or less than *V*_RE_ for the rapid redox reaction of PB according to the cyclic voltammogram of the lactate sensor electrode (Fig. 2e). The present difference of *V*_WE_ − *V*_RE_ = 0.13 V was higher than the reduction potential of 0.07 V of the PB mediator. This is because the difference caused between the cases with a commercial potentiostat and the developed organic circuit system. To check the consistency of the results in the two cases, the amperometric responses of the lactate sensor were measured at a potential of 0.13 V vs. Ag/AgCl using a commercial potentiostat (Fig. 4d). The sensitivity of 0.85 µA/mM was obtained from a commercial potentiostat (at a potential = 0.13 V vs. Ag/AgCl), which was approximately equivalent to that from the developed organic circuit system, as shown in Fig. 4e. In addition, when the mismatch between *V*_WE_ and *V*_RE_ in the developed system was elaboratively reduced to zero by tuning *V*_C_ of the inverter of the feedback control unit (see Supplementary Figure S4), the sensitivity of the lactate sensor was improved to the same level in Fig. 2f (at a potential = 0 V vs. Ag/AgCl) as shown in Figure 4f. It also improved the response time from a hundred seconds to several tens of seconds. These results indicate that the variation in *V*_M_ of the inverters should be minimized for further improving of the reproducibility, accuracy, and response time of the amperometric sensing system.

However, a major issue in printed OTFTs is the relatively large variations in their electrical properties^12,13^. Towards reducing variations in device performances, the formation process for organic semiconducting layers should be reconsidered along with that for uniform channel dimensions. Thus far, by employing the inkjet-printed electrodes and dispenser-printed banks (Fig. 3a), the standard deviation of the channel width and length were ±8 µm and ±2 µm, respectively. Furthermore, as a result of (1) controlling the DTBDT-C_6_ crystal growth direction^20^, (2) blending DTBDT-C_6_ and polystyrene^22^, and (3) annealing of the semiconducting layers, the standard deviation of the threshold voltage of the OTFTs was less than 0.03 V^23^. These methods mentioned above were also significant for improving the mobility or subthreshold slope (see supplementary Figures S5–S8). By employing these methods, the maximum variation in *V*_M_ of the inverters in this work was 0.15 V^23^, which is relatively small in the field of printed electronics. Nevertheless, further improvement of uniformity in *V*_M_ is still required. If the difference in *V*_M_ can be reduced to less than several tens of mV, the organic semiconductor devices will be acceptable for their potential applications to amperometric electrochemical sensing.

## Conclusion

In conclusion, we have developed a novel flexible and printed organic circuit system based on two negative-feedback inverters for wearable amperometric sensors with a three-electrode cell. The inverter was a pseudo-CMOS design for rail-to-rail operation and low output impedance, and consisted of only p-channel OTFTs employing a blend of a small molecular semiconductor, 2,7-dihexyl-dithieno[2,3-*d*;2′,3′-*d*′]benzo[1,2-*b*;4,5-*b*′]dithiophene, and polystyrene for the active layer as previously reported. The transimpedance amplifier based on the inverter with negative feedback exhibited a high linearity and tunable gain of 10^6^–10^7^ V/A. Input voltage was maintained at switching voltage of the inverter (*V*_M_) in the linear regime of output voltage. For a wide range of input current, reduction of the output impedance of the inverter should be required. A lactate sensor with an enzymatic membrane was used as the amperometric sensor. Quantitative measurement of lactate was successfully demonstrated by the developed system, showing the response time of a hundred seconds and the sensitivity of 1 V/mM at lactate concentration of 0–0.5 mM. The reduction of the variation in *V*_M_ to several tens of mV was found to be a significant requirement for maximizing the performance (sensitivity and response time) of the amperometric sensor with a prussian blue mediator, which is still an open question. Satisfying these requirements, namely the reduction of output impedance and *V*_M_ variation, allow organic semiconductor devices to have potential for realizing extremely thin and lightweight wearable devices for *in situ* monitoring of metabolites in body fluids.

## Methods

### Materials and Preparation of the Chitosan and Lactate Oxidase Solution

Chitosan solution (0.1 wt%, pH 5.4) was prepared by dissolving chitosan (Junsei Chemical) in HCl aqueous solution and stirring for 30 minutes. Lactate oxidase solution (1.0 UN/µl) was prepared by dissolving lactate oxidase (Toyobo, 85.6 UN/mg) in phosphate-buffered saline (Nacalai Tesque, 0.1 M, pH 7.4). The solutions were sealed and stored at 4 °C.

### Fabrication of the Lactate Sensors

125-µm-thick polyethylene naphthalate (PEN) films (Teijin, Teonex) were used as substrates without cleaning process. A 100-nm-thick Ag electrode was formed by inkjet-printing a silver nanoparticle ink (Harima Chemicals, NPS-JL) on the PEN substrates, followed by an annealing process of 120 °C for 30 minutes in the air. Then, a carbon graphite ink including prussian blue (Gwent Group) was coated on the printed Ag electrode, followed by an annealing process of 60 °C for 30 minutes in the air. In order to define the sensing area, a fluoropolymer solution (5 wt%, DuPont, Teflon AF1600) in Fluorinert (3M, FC-43) was printed as a bank onto the substrate except the sensing area by a dispenser, followed by an annealing process of 60 °C for 30 minutes in the air. 10 µl of the chitosan solution and 1.4 µl of the lactate solution was mixed. Then, 10 µl of the mixed solution was drop-casted onto the area defined by the fluoropolymer bank, followed by a drying process of 30 °C for 3 h in the air. The sensor electrodes were dipped in phosphate-buffered saline (PBS) and stored at 4 °C.

### Fabrication of the Organic Semiconductor Devices

The process flow is shown in Supplementary Figure S9. 125-µm-thick polyethylene naphthalate 125-µm-thick polyethylene naphthalate (PEN) films (Teonex, Teijin) were used as substrates without cleaning process. A silver nanoparticle ink in hydrocarbon-based solution (NPS-JL, Harima Chemicals) was printed as gate electrodes using an inkjet printer (Dimatix DMP2831, Fujifilm) with 10 pL nozzles. During the inkjet printing process, the substrates and cartridge were kept at 50 and 35 °C, respectively. The substrates were then heated at 120 °C for 30 minutes in the air to sinter the silver nanoparticles. A 150-nm-thick parylene (KISCO, diX-SR) gate dielectric layer was then formed by chemical vapor deposition. Source and drain electrodes were subsequently printed and sintered in the same manner as the gate electrodes. Fluoropolymer (1 wt%, Teflon AF1600, DuPont) in Fluorinert (FC-43, 3M) bank layers (200 nm thick) were then printed using a dispenser system (Image Master 350 PC, MUSASHI Engineering) at a pattering speed of 20 mm s^−1^ and with a discharge pressure of 6 kPa. During the dispensing process, the plate and nozzle temperatures were kept at 60 and 30 °C, respectively. To apply the self-assembled monolayer (SAM) treatment to source and drain electrodes, the substrates were immersed in a 3 × 10^−2^ mol/L 2-propanol solution of pentafluorobenzenethiol (Tokyo Chemical Industry) for 5 minutes at room temperature and rinsed with pure 2-propanol. The SAM treatment changed the work function of the printed silver electrodes from 4.7 to 5.4 eV, which reduces the contact resistance. A solution of DTBDT-C_6_ (0.9 wt%, Tosoh) and polystyrene (0.3 wt%, *M*_W_ ≈ 280,000, Sigma-Aldrich) in toluene was then printed onto the area defined by the bank layer by the dispenser system at a patterning speed of 20 mm s^−1^ and discharge pressure of 1 kPa, while keeping the stage and nozzle temperatures at 30 °C, followed by an anneal at 100 °C in the air for 15 minutes to remove the solvent. Finally, an encapsulation layer of Teflon was printed by the dispenser system at 30 °C, with a pattering speed of 8 mm s^−1^ with a discharge pressure of 6 kPa. The substrates were stored at room temperature in the air for three hours to remove the solvent.

### Characterization of the Lactate Sensors

The amperometric measurements were carried out using an electrochemical analyzer (BAS, model ALS612E). Before the measurement, the sensor electrode was soaked in PBS for at least 1 hour. During the measurements, the subject solution was stirred at 400 rpm.

### Characterization of the Organic Semiconductor Devices

The capacitance of the dielectric was measured using an LCR meter (NF, ZM2376). The electrical characteristics of the OTFTs and inverter circuits were measured using a semiconductor parameter analyzer (Keithley, model 4200A-SCS). All electrical measurements were carried out in the air. Optical microscope images of the devices were obtained using a digital microscope (Keyence, model VHX-5000).

### Measurement of Lactate with the Organic Circuit System

Voltage supplying and measurement was carried out using a semiconductor parameter analyzer (Keithley, model 4200A-SCS). The inverters were connected with a resistance, a reference electrode (Ag/AgCl), counter electrode (Platinum) and a lactate sensor electrode via coaxial cables covered with aluminum foil to reduce noise. The conversion system was biased (*V*_C_ = 3 V, *V*_DD_ = 4 V) for 5 minutes before the measurement to stabilize the operation.

## Electronic supplementary material

Supplementary information
