# Supplementary material for: A Printed Organic Circuit System for Wearable Amperometric Electrochemical Sensors
Source: Sci Rep. 2018 Apr 23;8:6368. doi: 10.1038/s41598-018-24744-x (PMC5913266; doi:10.1038/s41598-018-24744-x)
Supplement: Supplementary file 1 — Supplementary information [file 41598_2018_24744_MOESM1_ESM.pdf]

Supplementary information

A Printed Organic Circuit System for Wearable Amperometric Electrochemical Sensors

Rei Shiwaku[1], Hiroyuki Matsui[1,a], Kuniaki Nagamine[1,a], Mayu Uematsu[1], Taisei Mano[1], Yuki Maruyama[1], Ayako Nomura[1], Kazuhiko Tsuchiya[1], Kazuma Hayasaka[1], Yasunori Takeda[1], Takashi Fukuda[2], Daisuke Kumaki[1], and Shizuo Tokito[1,a]

[1]Research Center for Organic Electronics (ROEL), Yamagata University, 4-3-16 Jonan, Yonezawa, Yamagata, 992-8510, Japan

[2]Functional Polymers Research Laboratory, Tosoh Corporation, 1-8 Kasumi, Yokkaichi, Mie, 510-8540, Japan

[a]Author to whom correspondence should be addressed: h-matsui@yz.yamagata-u.ac.jp (H. Matsui), nagamine@yz.yamagata-u.ac.jp (K. Nagamine), tokito@yz.yamagata-u.ac.jp (S. Tokito)

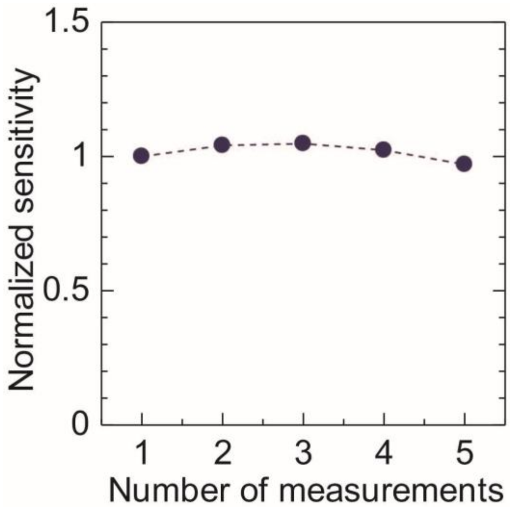

Figure S1. Normalized sensitivity of the lactate sensor under five repetitive measurements. Change of the sensitivity was less than 5%.

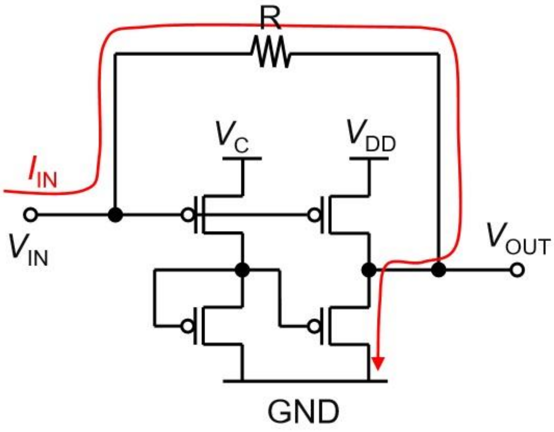

Figure S2. Circuit diagram of the transimpedance amplifier of the detection unit using a pseudo-CMOS inverter. The major path of the current from the input terminal is indicated by a red arrow.

Here, the relation between V_{OUT} and I_{IN} is shown below. Taking the voltage drops at each of the sections into consideration, following equations can be obtained:

$$V_{OUT} - V_M = -A_{open}(V_{IN} - V_M), \text{ (S1)}$$

$$I_{IN} = \frac{V_{IN} - V_{OUT}}{R}, \text{ (S2)}$$

where V_M and A_{open} is the switching voltage and the open-loop gain of the inverter, respectively. Combining the equation (S1) and (S2) to remove V_{IN}, V_{OUT} is expressed as:

$$V_{OUT} = V_M - \frac{1}{1 + \frac{1}{A_{open}}} RI_{IN}, \text{ (S3)}$$

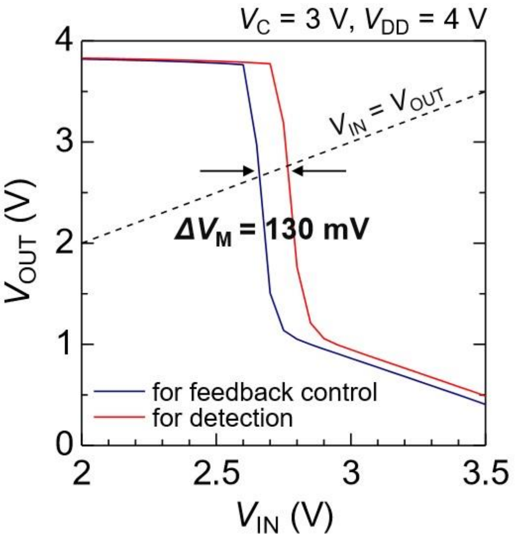

Figure S3. Input-output characteristics of the two inverters on the same substrate.

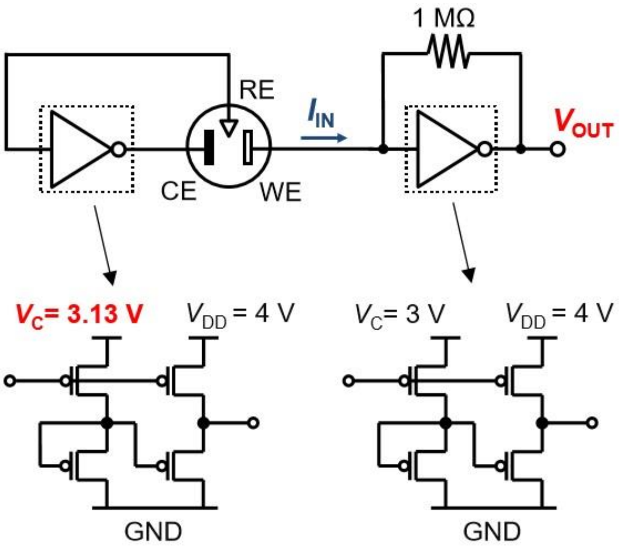

Figure S4. Setting of the supply voltage of the organic circuit system for no potential difference between the reference and working electrode. V_C of the feedback control inverter and detection inverter was set to 3.13 V and 3 V, respectively.

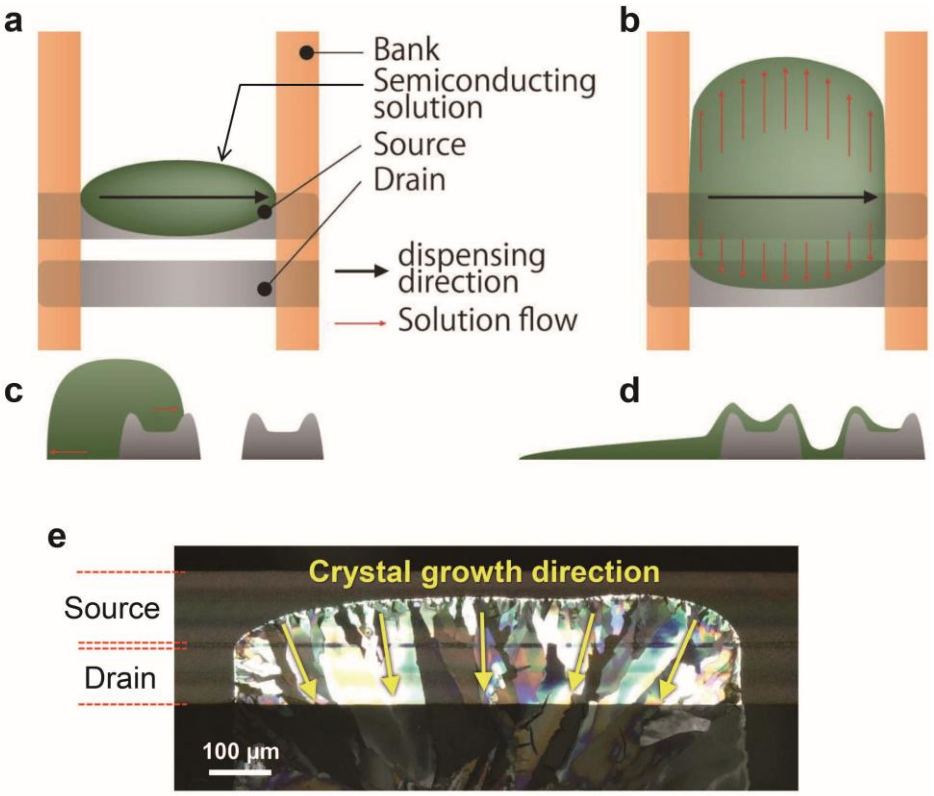

Figure S5. Method for controlling the crystal growth direction. Schematic topview (a) before and (b) after semicondctor solution spreads**.** Cross-sectional view (c) before and (d) after semicondctor solution spreads**.** (e) Photograph of a resulting semiconductor crystal.

After the DTBDT-C_6 semiconductor solution was deposited onto the substrates, the solution spread and DTBDT-C_6 crystals grew from the periphery of the deposited droplet to the center. The off-center areas of the semiconducting layer, where the DTBDT-C_6 crystals grew in one direction, was used as the transistor channel regions in order to enhance OTFT electrical performance and uniformity.

For more details, see Ref. 20, Fukuda, K. *et al*. Printed organic transistors with uniform electrical performance and their application to amplifier in biosensors. *Adv. Electron. Mater.* **1**, 1400052 (2015).

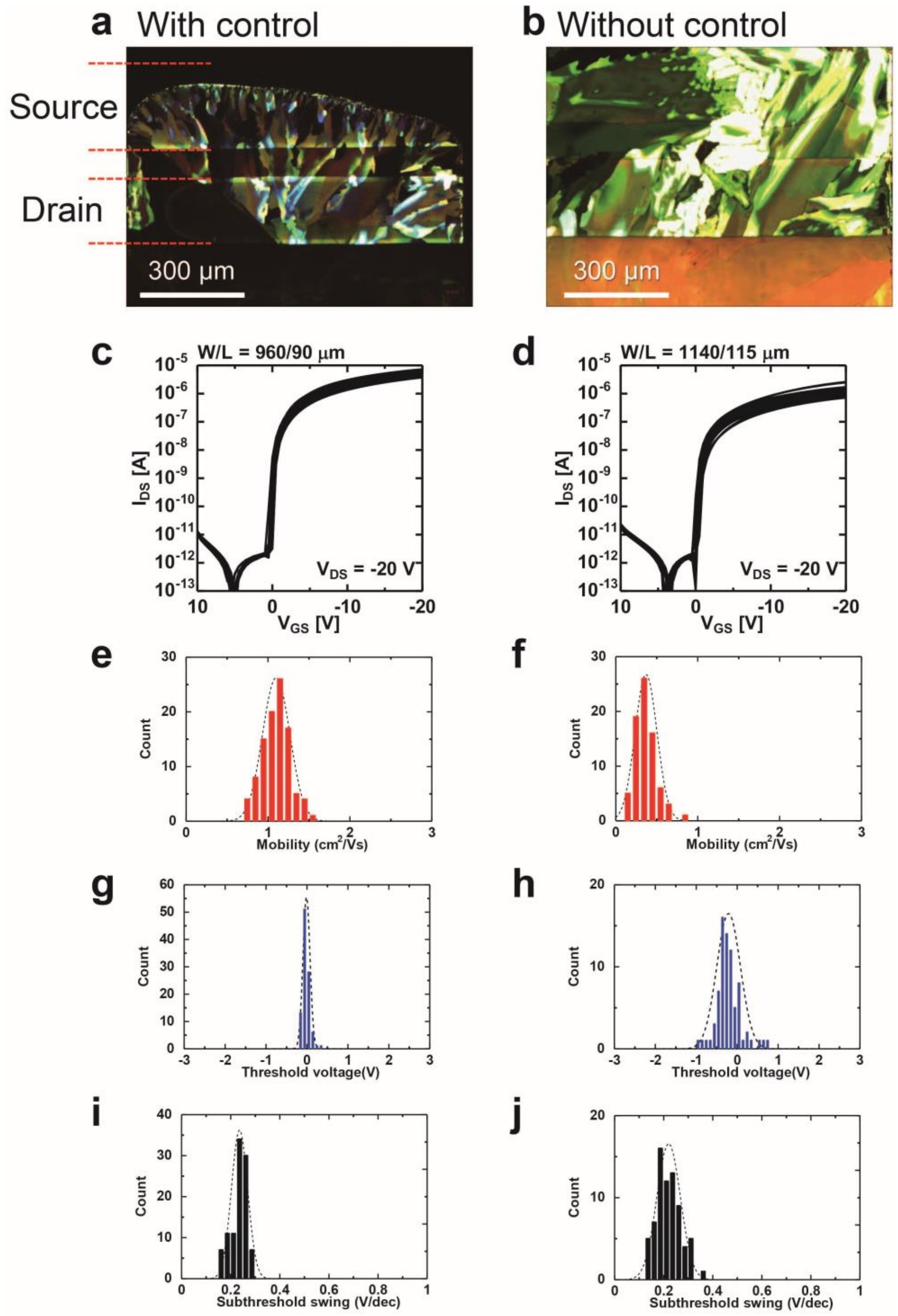

Figure S6. Effect of controlling the crystal growth direction on the device performances. 1.0-wt%-DTBDT-C_6 in toluene was used as the semiconductor ink. Photograph of the OTFT channel (a) with control (dispenser printing) and the OTFT channel (b) without control (drop-casting). Transfer characteristics of OTFTs (c) with control (100 devices) and (d) without control (72 devices). Distribution of the mobility for the OTFTs (e) with control (1.1 ± 0.17 cm^2/Vs) and (f) without control (0.4 ± 0.13 cm^2/Vs). Distribution of the threshold voltage for the OTFTs (g) with control (−0.01 ± 0.09 V) and (h) without control (−0.21 ± 0.29 V). Distribution of the subthreshold slope for the OTFTs (*SS*) (i) with control (0.24 ± 0.03 V/dec) and (j) without control (0.22 ± 0.05 V/dec).

For more details, see Ref. 20, Fukuda, K. *et al*. Printed organic transistors with uniform electrical performance and their application to amplifier in biosensors. *Adv. Electron. Mater.* **1**, 1400052 (2015).

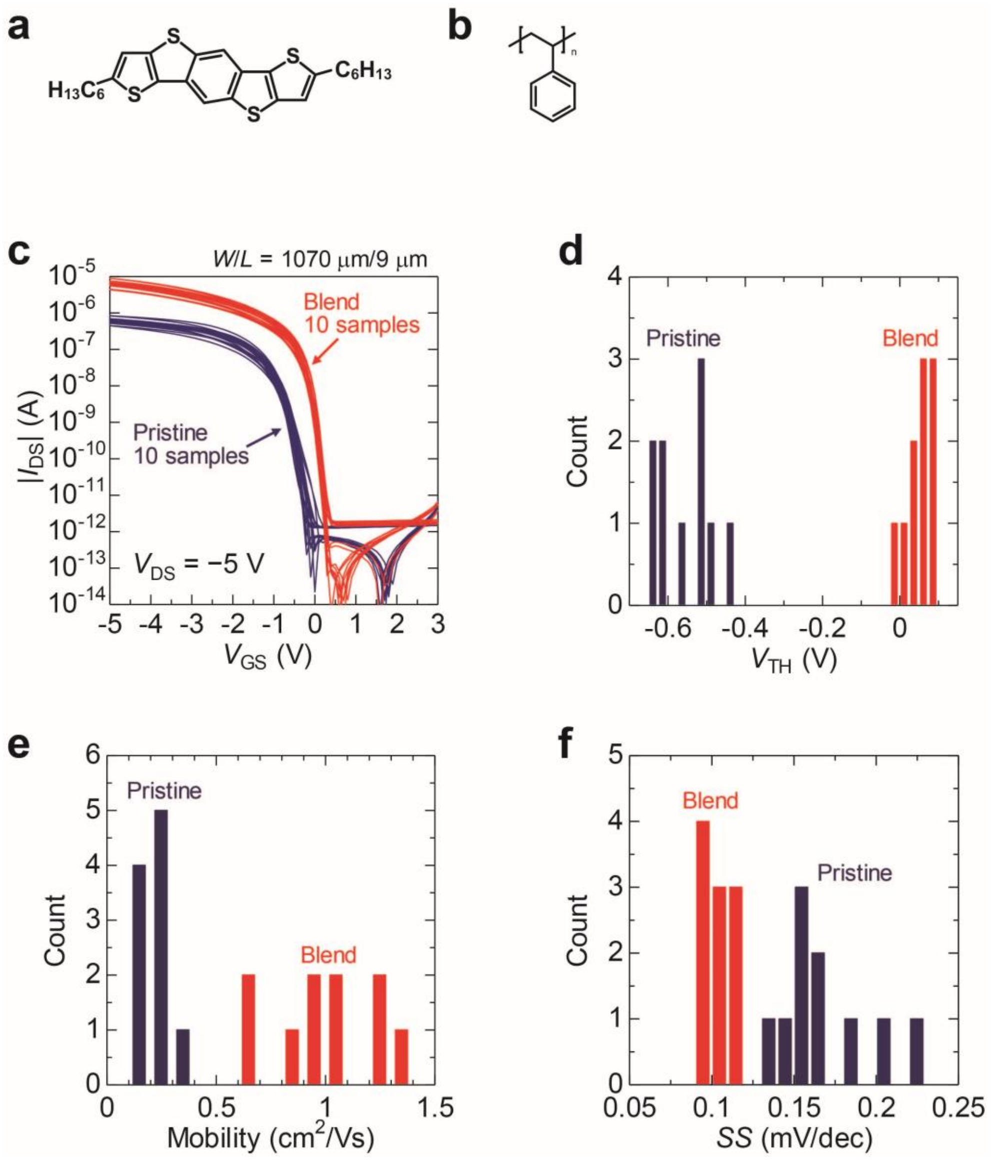

Figure S7. Effects of blending DTBDT-C_6 and polystyrene on the device performances. Chemical structure of (a) DTBDT-C_6 and (b) polystyrene (PS). The mass ratio of the DTBDT-C_6:PS blend ink was 4:1. An annealing treatment after formations of the organic semiconducting layer was not applied. (c) Transfer characteristics of the DTBDT-C_6:PS blend TFTs (red line) and the pristine DTBDT-C_6 TFTs (indigo line). (d) Distribution of threshold voltage (V_{TH}) for the blend (0.05 ± 0.04 V) and the pristine (−0.55 ± 0.07 V). (e) Distribution of mobility for the blend (1.0 ± 0.2 cm^2/Vs) and the pristine (0.22 ± 0.06 cm^2/Vs). (f) Distribution of subthreshold slope (*SS*) for the blend (0.099 ± 0.008 V/dec) and the pristine (0.164 ± 0.027 V/dec).

For more details, see Ref. 22, Shiwaku, R. *et al*. Printed 2 V-operating organic ivnerter arrays employing a small-molecule/polymer blend. *Sci. Rep.* **6,** 34723 (2016).

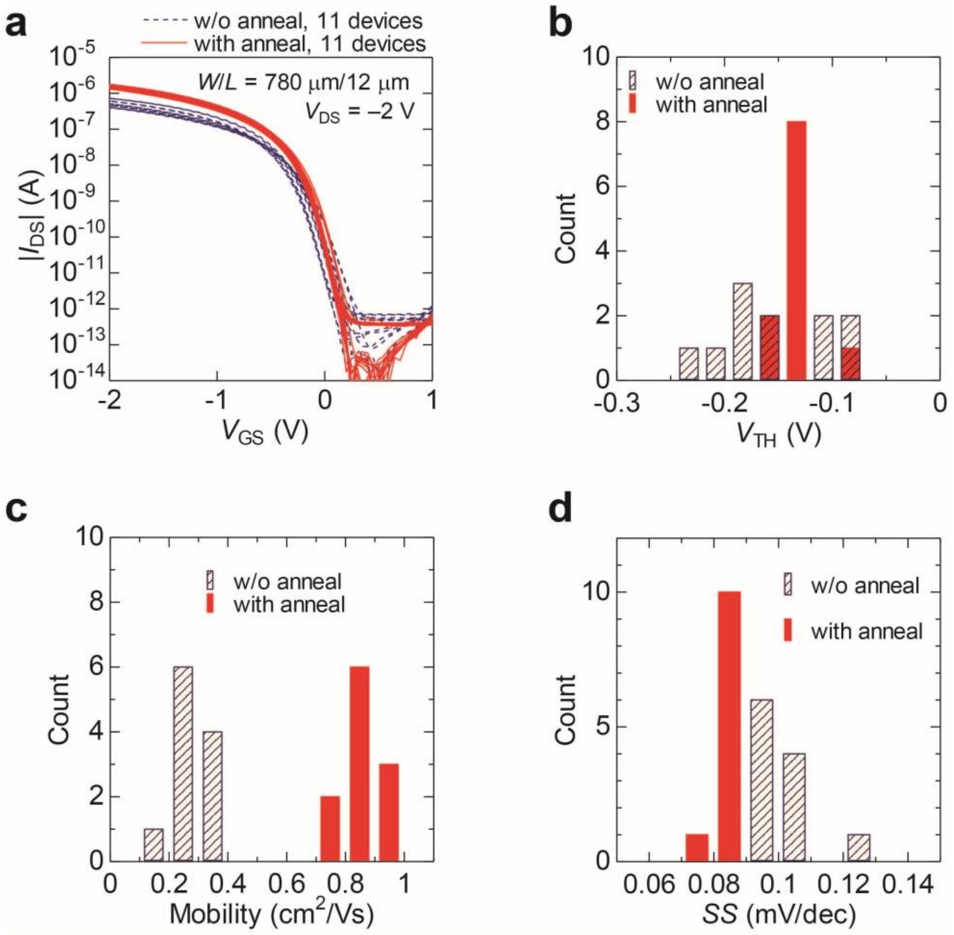

Figure S8. Effects of annealing treatment for the DTBDT-C_6:PS blend semiconducting layer on the device performances. The mass ratio of the DTBDT-C_6:PS blend ink was 3:1. (a) Transfer characteristics of the devices which was annealed at 100 °C for 15 min. in air ambient (dashed blue line) and not annealed (30 °C for 15 min. in air ambient, solid red line) after formations of the organic semiconducting layer. (b) Distribution of threshold voltage (V_{TH}) for the devices with anneal (−0.13 ± 0.02 V) and the devices without anneal (−0.15 ± 0.05 V). (c) Distribution of mobility for the devices with anneal (0.85 ± 0.07 cm^2/Vs) and the devices without anneal (0.27 ± 0.05 cm^2/Vs). (d) Distribution of subthreshold slope (*SS*) for the devices with anneal (0.080 ± 0.002 V/dec) and the devices without anneal (0.100 ± 0.006 V/dec).

For more details, see Ref. 23, Shiwaku, R. *et al*. Printed organic inverter circuits with ultralow operating voltages. *Adv. Electron. Mater.* **3,** 1600557 (2017).

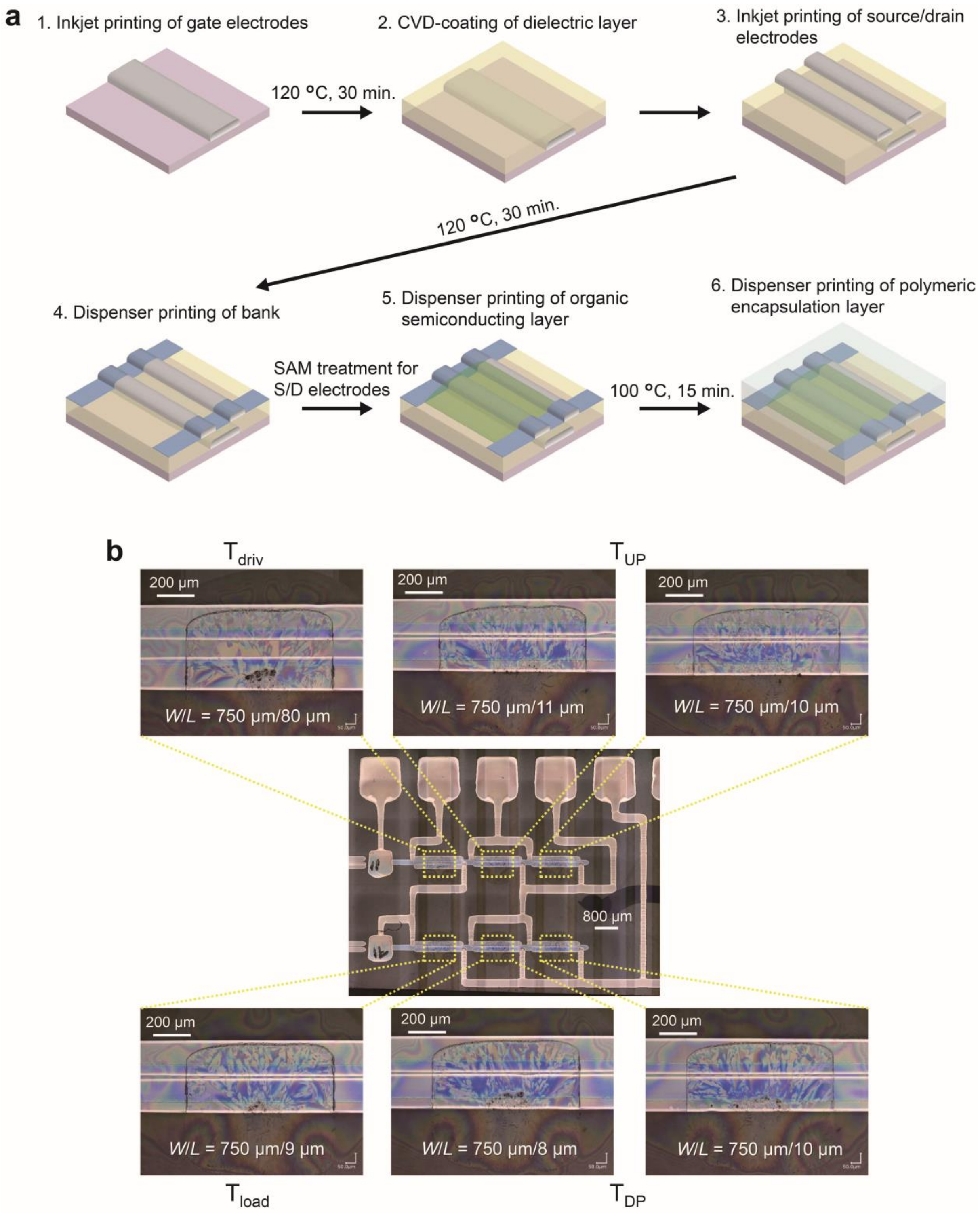

Figure S9. Process flow for the fabrication of the printed OTFTs and circuits. (a) Process flow for the fabrication of the organic semiconductor devices. Process flow for the circuits was same as that for the OTFTs. (b) Photographs of the OTFTs and circuit fabricated via the process above.
